# Advancing microfluidic point-of-care platelet function tests: opportunities and challenges from bench to market

**DOI:** 10.3389/fbioe.2024.1507972

**Published:** 2024-12-16

**Authors:** Minki Kang, Christopher A. Bresette, David N. Ku

**Affiliations:** George W. Woodruff School of Mechanical Engineering, Georgia Institute of Technology, Atlanta, GA, United States

**Keywords:** platelet function testing, microfluidic, point of care, shear-induced platelet aggregation, diagnostics

## Abstract

Platelets are critical for blood clotting, with shear-induced platelet aggregation (SIPA) playing a key role in hemostasis and the prevention of excessive bleeding. SIPA function potentially leads to life-threatening diseases such as hemorrhage and myocardial infarction, which are leading causes of death globally. Point-of-care platelet function tests (POC PFTs) are developed to assess platelet dysfunction and distinguish between normal and abnormal platelet activity. Recent advances in microfluidic technology have been integrated into POC PFTs, showing promise for delivering more accurate, rapid, and differentiated results using minimal blood sample volumes, enabling more informed treatment decisions. However, current POC PFTs fall short of replicating high-shear thrombotic conditions *in vitro*, resulting in limited clinical SIPA diagnosis and actionable insights. In this review, we explore the current landscape of POC PFT technology, key challenges, and future opportunities. We highlight the importance of device design and scalable manufacturing to fully realize the potential of microfluidic POC PFTs and facilitate their widespread adoption in clinical practice, ultimately improving patient outcomes.

## 1 Introduction

Platelets are essential for both controlling bleeding and facilitating wound repair, acting as key regulators of hemostasis through shear-induced platelet aggregation (SIPA) (Casa and Ku, 2017; Liu et al., 2022). SIPA is an independent mechanochemical phenomenon that develops at high shear flow over 5,000 s^−1^, which involves elongation of von Willebrand factor (vWF), and aggregation with platelets at the thrombotic surface (Reininger, 2006; Hansen et al., 2011; Colace and Scott, 2013; Rana et al., 2019). The mechanisms behind SIPA are finely balanced to stop bleeding while maintaining normal blood flow within the circulatory system. Abnormalities in SIPA function can lead to a wide range of disorders, from mild clotting abnormalities to severe, life-threatening conditions such as hemorrhage, thrombosis, or acute myocardial infarction. In the United States, over 356,000 people experience out-of-hospital cardiac arrests each year, with mortality rates close to 90%, even with advanced paramedic interventions (What Is Sudden Cardiac Death, 2023; Heart Attack and Sudden Cardiac Arrest Differences, 2022). Hemorrhage, often resulting from impaired platelet-mediated hemostasis due to trauma, postpartum complications, or underlying medical conditions, causes over 60,000 deaths annually, with around 25% of these fatalities linked to compromised platelet function(Latif et al., 2023).

The medical and societal importance of assessing platelet dysfunction has driven the development of point-of-care platelet function tests (POC PFTs) (Al et al., 2019; Sharan et al., 2019; Mammen et al., 2024). Over recent decades, PFTs have advanced from traditional qualitative tests to quantitative methods that partially replicate *in vivo* thrombotic conditions (Le Blanc et al., 2020; Yoon et al., 2024). Early diagnostics, such as bleeding time measurements from the 1960s and static thromboelastography (TEG^®^) for coagulation profiling, have evolved into assays like static platelet reactivity turbidimetry (VerifyNow^®^), multiple electrode platelet aggregometry (Multiplate Analyzer), and the platelet function analyzer (PFA-100/200). Such conventional PFTs provide different metrics for platelet aggregation in various ways. TEG^®^ and VerifyNow^®^ assess the coagulation affinity of a patient’s blood under static conditions. Light Transmission Aggregometry (LTA) measures the changes in light transmission by platelet aggregates. The PFA-100 measures the time required for a sample of whole blood to occlude an orifice in an agonist-coated membrane. The growing demand for precise diagnostic tools in clinical settings has fueled significant growth in the PFT market. The global PFT market, valued at USD 1.2 billion in 2022, is expected to reach USD 2 billion by 2030, with a compound annual growth rate (CAGR) of 6.8% (“Platelet Function Testing Market Size, Demand and Challenges By 2030” 2022). This growth is largely due to the rising prevalence of cardiovascular diseases, bleeding disorders, and the increasing demand for personalized medicine in managing thrombosis and hemostasis. As life expectancy rises and platelet-related disorders become more recognized, especially in women and the elderly, the demand for PFTs is likely to grow. Favorable regulatory developments, such as Sysmex Corporation’s 2015 approval to manufacture platelet aggregation agonist reagent kits for *in vitro* diagnostics, have also spurred market expansion.

Thorough replication of high shear rate flow and thrombotic surface *in vitro* is crucial to provide an accurate assessment of SIPA. Significant research efforts have been dedicated to developing miniaturized, microfluidic devices that simulate physiological high-shear thrombotic conditions (Hosokawa et al., 2011; Hastings et al., 2017; Zhang et al., 2021; Zhao et al., 2021). Compared to static systems like VerifyNow^®^ or TEG^®^, microfluidic devices hold the potential to create high shear rate flows with small blood samples. Point-of-Care (POC) designs offer rapid endpoints for a SIPA assay at the bedside, aligning with broader healthcare goals of improving patient outcomes while reducing costs. However, key challenges remain, such as the need for optimized flow designs, scalable manufacturing, and high precision to fully unlock the potential of POC PFTs.

This review explores the opportunities for improving microfluidic POC PFTs and identifies key design requirements and challenges to facilitate their commercialization. By examining the current landscape of PFTs, with a focus on the advances and potential of microfluidic systems, we highlight critical determinants of thrombosis and potential applications in clinical settings and biomedical research. Additionally, it outlines design requirements and recent progress in microfluidic systems. A comparative analysis of existing POC PFTs is provided, with an emphasis on assessing platelet function in relation to SIPA. Lastly, we address major challenges in advancing these technologies, such as scalable manufacturing, diagnostic reliability, accurate replication, and indirect diagnostic information. This review offers purpose-oriented guidelines for developing effective and clinically relevant POC PFTs.

## 2 Current commercially available POC PFTs

**FIGURE 1:**
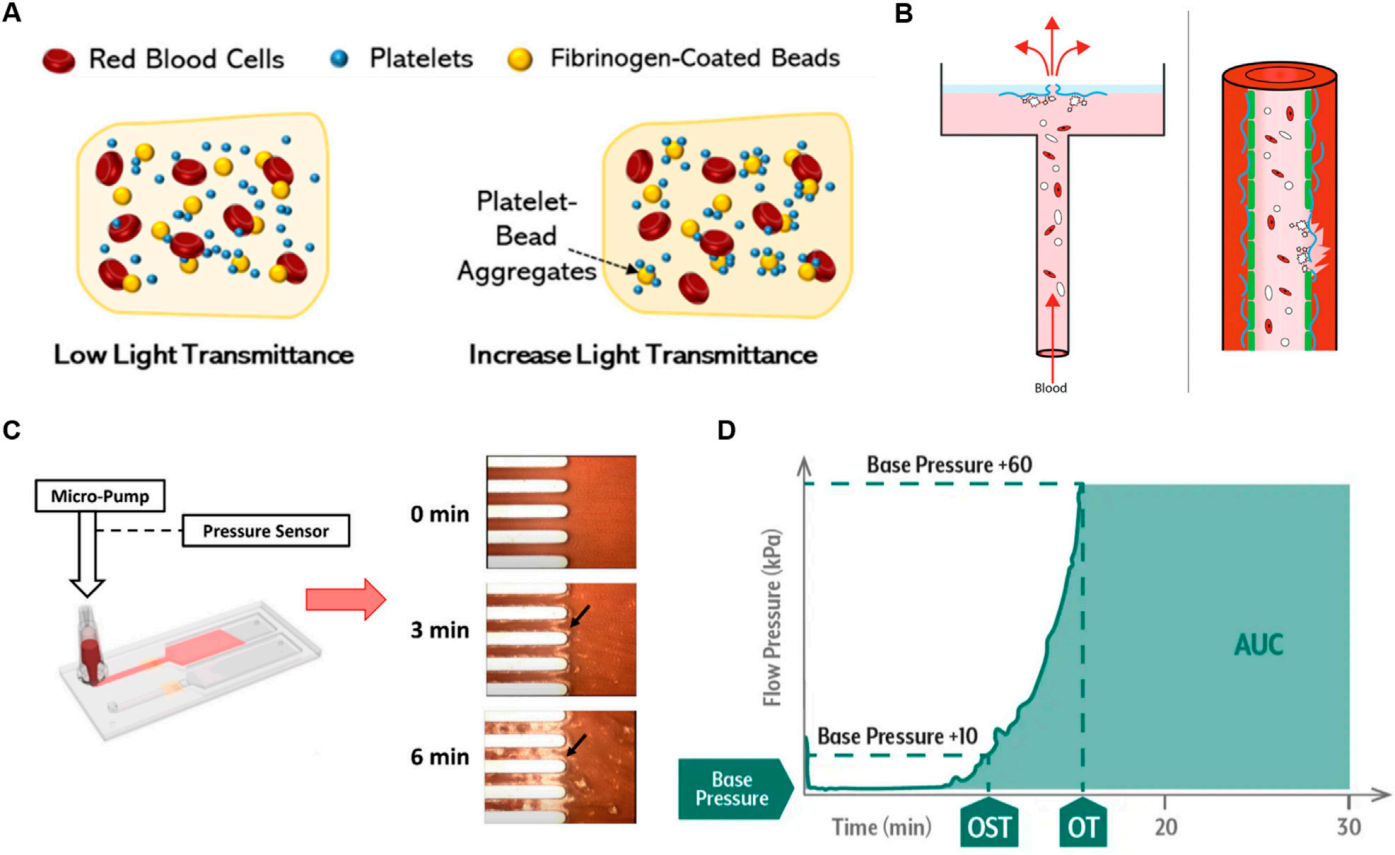
Overview of Currently Used Platelet Function Testing POCs **(A)** Schematic of VerifyNow^®^ and LTA working principles, demonstrating platelet aggregation detection in response to agonists. Reprinted from Yoon et al. (2024), Copyright 2024, with permission from MDPI. **(B)** Platelet aggregation data measured by the born PFA^®^ in response to agonists. Reprinted from Van Oosterom et al. (2022), Copyright 2021, with permission from Taylor and Francis Group. **(C)** Working principle of T-TAS with microscopy images showing thrombus growth in microchannels. **(D)** Example T-TAS data plotting flow pressure versus time, with the endpoint marked by the Area Under the Curve (AUC) (OST: occlusion start time, OT: occlusion time). Reproduced with permission from Fujimori Kogyo, Co. Ltd., Tokyo, Japan.

Figure 1 illustrates schematic device design and working principles of currently used POC PFTs and microfluidic systems and Table 1 provides comparative features, pros, and cons. Bleeding time tests may be representative of SIPA. Other platelet assays include LTA and the VerifyNow^®^ system that are clinically used for platelet function testing, quantifying the aggregation capacity of platelets in response to various agonists such as Adenosine DiPhosphate (ADP), collagen, or epinephrine (Figure 1A) (Yoon et al., 2024; Cattaneo, 2009). LTA measures the optical changes in Platelet-Rich Plasma (PRP) as platelets aggregate. Initially, PRP has high light transmission due to light scattering caused by the suspended platelets. When an agonist is added, the platelets activate and begin to clump together, decreasing the turbidity of the sample. (Dichiara et al., 2007). As the platelet aggregates grow, they fall out of suspension, increasing light transmittance. This change in light transmission generates a detailed trace that characterizes platelet function and generalized platelet reactivity in patients treated with drugs such as aspirin. They are widely adopted to assess platelet function, however, many traditional tests, such as VerifyNow^®^, Plateletworks, and TEG^®^, do not involve blood flow, do not replicate high shear platelet activity, thus they do not assess SIPA. (Wang et al., 2018).

**TABLE 1:** Comparative analysis of currently used POC PFT instruments.

Instrument	Manufacturer	Technique	Anticoagulant/Agonist	Shear (s^-1^)	Advantages	Disadvantages
VerifyNow^®^ (Dichiara et al., 2007)	Werfen, Barcelona, Spain	LTA through whole blood which depends on the degree of platelet aggregation around fibrinogen coated beads	Citrate/AA, ADP, GPIIb/IIIa	No flow	Whole blood test, User-comfort, simple and rapid result	Lack of high shear flow, does not assess SIPA
PFA-100 PFA-200 (Mammen et al., 2024; Moenen et al., 2019)	Siemens Healthcare GmbH, Erlangen, Germany	Measure occlusion time after perfusing whole blood	Citrate/Collagen-EPI, Collagen-ADP, Innovance P2Y	∼5,000 (aspiration)	Relevance to SIPA, Whole blood test, small blood consumption, simple and rapid test results for severe platelet defects	Non-physiological flow due to fixed initial shear rate, soluble exogenous agonists
Plateletworks (Gorog and Fuster, 2013; Gorog and Becker, 2021)	Helena Laboratories, Beaumont, Texas, United States	Measures platelet count before and after the addition of platelet agonist	EDTA (References tube)/ADP, Collagen, AA	No flow	Low cost, small blood sample volume, simple and rapid	Indirect test, scarce data, does not assess SIPA
TEG^®^ (Le Blanc et al., 2020; Gorog and Becker, 2021)	Haemonetics Corporation, Brain-tree, MA, United States	Measurement of whole blood clotting (not platelet function), in response to rotation and measurement of viscoelastic clot characteristics reflected by changes in impedance	Citrate/kaolin, ellagic acid, tissue factor	No flow	Real-time monitoring	Measures coagulation clot mechanics. Does not assess SIPA or platelet function
Global thrombosis test (Wang et al., 2018)	Thromboquest Ltd., London, United Kingdom	Measurement of thrombus formation in response to high shear, and subsequent measurement of spontaneous thrombolysis	-	∼12,000 s^-1^	Whole blood test, no citrate, high shear rates, small blood sample volume, simple interpretation	Short testing interval, inconsistent results, variety of thrombogenic surfaces, no commercial availability
T-TAS (Kaikita et al., 2019; Al et al., 2019)	Fujimori Kogyo Co. Ltd., Tokyo, Japan	Measurement of the area under the flow pressure curve (AUC) perfusing whole blood	PL chip; Hirudin or BAPA/type I collagen AR chip; citrate/type I collagen, tissue thromboplastin	PL chip; 1,500 s^-1^ AR chip; 600 s^-1^ HD chip; 1,200 s^-1^	Global assay, Arterial flow conditions, small blood consumption, whole blood sample, comprehensive analysis of primary hemostasis	High noise in curve and variability due to the use of constant flow, use of citrate, lack of differentiation ability
Microfluidic POCs (van Rooij et al., 2020)	-	Measurement of occlusion time and blood volume	Type I collagen	2000–12,000 s^-1^	Small blood consumption, whole blood sample, comprehensive analysis of primary hemostasis, low noise level, high reliability	Lack of scalable manufacturing, high variability in outcomes, and careful flow management required

The platelet function analyzer (PFA-100) provides a quantitative metric of closure time which is the interval required for platelets to form a plug and occlude a membrane. Such systems were marketed as a global assay for platelet-dependent primary hemostasis (Figure 1B) (Moenen et al., 2019; Oosterom et al., 2022; Mammen et al., 2024). Citrated whole blood is aspirated through a capillary into a central orifice of a bioactive membrane, the surface of which is coated with thrombogenic collagen, in some cases agonists such as ADP. Similarly, T-TAS is a microfluidic chip-based PFT that assesses platelet thrombus formation under physiological shear flow conditions up to 1,500 s^-1^ (Figures 1C, D). (Jain et al., 2016; Kaikita et al., 2019; Al et al., 2019) The T-TAS platform has three microchips; one of them, the PL chip is FDA-cleared for use in the clinic, and others are for research use only. The PL chip incorporates 26 microfluidic capillary channels coated with Type I collagen and is designed to detect defects in primary hemostasis by measuring the increase in flow pressure caused by occlusion. The AR chip and HD chip, whose test sections are coated with collagen and tissue thromboplastin, assess fibrin-rich thrombus formation by activating both platelets and the coagulation system. The HD chip works with whole blood samples, even at low platelet concentrations as low as 10 k·µL^-1^. Note that physiologic coagulation occurs under static conditions, not flow. Thrombus formation is monitored via upstream pressure changes while under flow control, with the area under the curve (AUC) used as a measure of thrombogenicity. The above systems partially replicate pathologic shear conditions and assess SIPA, however, they require a long testing period and may not provide consistent and accurate differentiation of abnormality due to the lack of a well-defined flow channel shear rate and thrombus growth. (Kaikita et al., 2019).

While soluble agonists released from platelets play a secondary role mainly in stabilizing formed aggregates, the most commonly used tests still employ soluble agonists such as ADP or collagen to initiate platelet aggregation. (Jackson, 2007; Nesbitt et al., 2009; Wang et al., 2018). Another main issue is the use of citrate as an anticoagulant. Most POC PFTs in current clinical use are performed on citrated blood except for the Global Thrombosis Test and Plateletworks. Despite citrate anticoagulation being a very convenient way to handle blood samples, allowing storage for a few hours, citrate has a dramatic effect on both platelet function and SIPA that is not physiological. (Baumgartner et al., 1980; Gilman et al., 2013; Silverberg et al., 2017). It lacks relevance to the function of whole blood and platelets under thrombotic or hemorrhagic conditions *in vivo*. Also, calcium fluctuations caused by citrate impact the binding of coagulation factors, leading to reduced thrombus stability and inaccurate platelet function testing results. Other possible anticoagulants include Heparin (Liu et al., 2022), EDTA, D-phenylalanyl-L-prolyl-L-arginine chloromethyl ketone dihydrochloride (PPACK) (Ruggeri et al., 2006), and Benzylsulfonyl-D-Arg-Pro-4-amidinobenzylamide (BAPA) (Kaikita et al., 2019).

## 3 Arterial thrombosis mechanisms and concept of microfluidic POC PFTs

**FIGURE 2:**
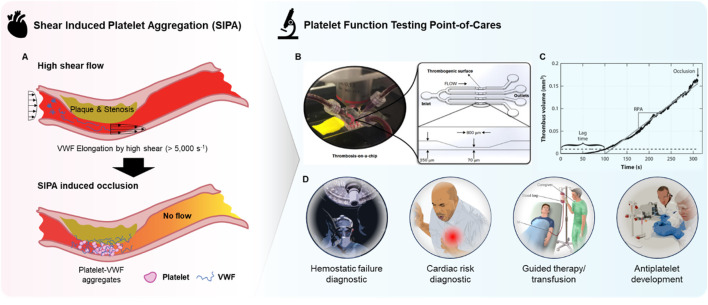
Overview of microfluidic POC PFTs. **(A)** Schematic representation of arterial thrombosis driven by SIPA, illustrating the process of shear-induced platelet aggregation under high shear rates. **(B)** Photograph of a microfluidic device and its corresponding microchannel design, simulating thrombosis-on-a-chip for the study of platelet function and thrombus formation. Reprinted from Liu et al. (2022), Copyright 2022, with permission from American Society of Hematology **(C)** Graph showing thrombus volume as a function of time, highlighting the progression of SIPA and the resulting occlusion in the microchannel. Reproduced with permission from Casa et al. (2015), Copyright 2015, with permission from Elsevier. **(D)** Overview of potential clinical and research applications of microfluidic POC PFTs.

The rapid growth of thrombi at sites of atherosclerotic stenosis or during hemostasis following vascular injury is driven by SIPA, primarily involving the mechanochemical interactions between VWF, platelets, and thrombogenic surfaces at high wall shear rates (WSR) above 5,000 s⁻^1^ (Figure 2A). SIPA is distinctly different from coagulation, as coagulation occurs under low shear flow or static conditions (Ruggeri et al., 2006; Casa et al., 2015; Gorog and Becker, 2021). The variable driving SIPA is shear rate and does not require a specific shear rate gradient. Shear gradient promotes platelet activation and thrombi stabilization, however, at pathological shear rates (>10,000 s^−1^) platelet aggregates can develop independently of them. (Ruggeri et al., 2006; Nesbitt et al., 2009). Under high shear conditions, VWF undergoes a conformational shift from a compact, globular form to an elongated structure (Casaet al., 2015). This change exposes more A1 domains on VWF, which act as binding sites for glycoprotein Ib (GPIb) on platelets (Reininger, 2006). Simultaneously, VWF adheres to thrombogenic surfaces such as the subendothelial collagen matrix. This leads to the rapid formation of VWF-platelet aggregates, which are captured on the vessel wall through VWF binding, followed by quick thrombus growth and eventual occlusion. While the αIIbβ3-fibrinogen bond supports platelet adhesion under low shear conditions (<100 s⁻^1^), SIPA in high shear flow relies on the rapid kinetics of bond formation between GPIb-A1. The GPIb-A1 bond forms with a rate between 10⁶ and 10⁹ M⁻^1^s⁻^1^, significantly faster than the ∼10⁴ M⁻^1^s⁻^1^″on-rate” of the αIIbβ3-fibrinogen bond (Liu et al., 2022). In summary, the SIPA is a biophysical phenomenon that requires a combination of three conditions; i) wall shear rate that is pathologically high, ii) the presence of sufficient platelets and VWF, and iii) a thrombogenic surface such as fibrillar collagen.

Microfluidic devices can utilize whole blood samples, stenosis regions where high-shear flow occurs due to narrowing geometry, and thrombotic surfaces that facilitate VWF adsorption and aggregate capture (Figure 2B). Such configuration fulfills the three SIPA requirements, sufficiently replicating arterial thrombosis *in vitro*. The primary benefits include the ability to form precisely controlled high shear flow with minimal blood consumption by leveraging a micrometer-scale flow channel (Gurkan et al., 2024). Additionally, thrombus growth and occlusion can be observed and quantified with endpoints such as occlusion time or occlusion end-volume, providing direct interpretation without the need for costly measurement tools (Figure 2C) (Kim et al., 2019). Furthermore, the combination of rapid thrombus growth and small channel features results in short testing times (<5 min) as shown in Figure 2C as thrombus can rapidly occlude the channel. These factors maximize the efficacy of microfluidic POC PFTs as diagnostic tools in clinical settings, where quick, accurate, direct, and actionable information is required with limited blood samples.

The ability to observe platelet responses under near-physiological conditions may allow for a more comprehensive evaluation of SIPA, which can be particularly useful for identifying patients at risk of hemostatic failure and cardiac risk, implementation of guided therapy or transfusion, or assessing the efficacy of antiplatelet therapies (Figure 2D) (Moenen et al., 2019; Rana et al., 2019; Oosterom et al., 2022; Gorog and Becker, 2021). Additionally, the speed at which microfluidic POC PFTs can deliver results is another key advantage, offering expedited data in several minutes that can be critical in time-sensitive clinical scenarios such as cardiovascular emergencies or surgical interventions. Microfluidic POC PFTs also have the potential to significantly improve the accessibility and efficiency of platelet function testing in clinical settings as point-of-care diagnostics, reducing the need for centralized laboratory testing and enabling rapid, bedside evaluations. This could lead to faster treatment decisions and improved patient outcomes, particularly in urgent care situations. Automation within these systems may further streamline platelet testing workflows, reducing the need for highly trained personnel and specialized laboratory equipment.

In addition to assaying the SIPA function of a patient’s blood, microfluidic POC PFTs can measure patient-specific responses to antiplatelet drugs and may be used to tailor treatment. One use of PFTs is in detecting individuals who are resistant to antiplatelet treatments, such as aspirin. (Kaikita et al., 2019). Aspirin resistance affects up to 27% of the population and can lead to excessive bleeding risks without the benefit of reducing thrombotic risk. (Gurbel et al., 2007). Similarly, PFTs may be used to determine the efficacy of antiplatelet medications after initiation of treatment. (Mammen et al., 2024; Kaikita et al., 2019). Finally, microfluidic POC devices are being used to search for and develop new anti-thrombotic drugs. (Bresette et al., 2024; Sakai et al., 2018). The benefits of using POC devices for drug discovery are their proven clinical translation, high throughput, ease and speed of use, and low blood volume requirements.

## 4 Recent progress in microfluidic POC PFTs

**FIGURE 3:**
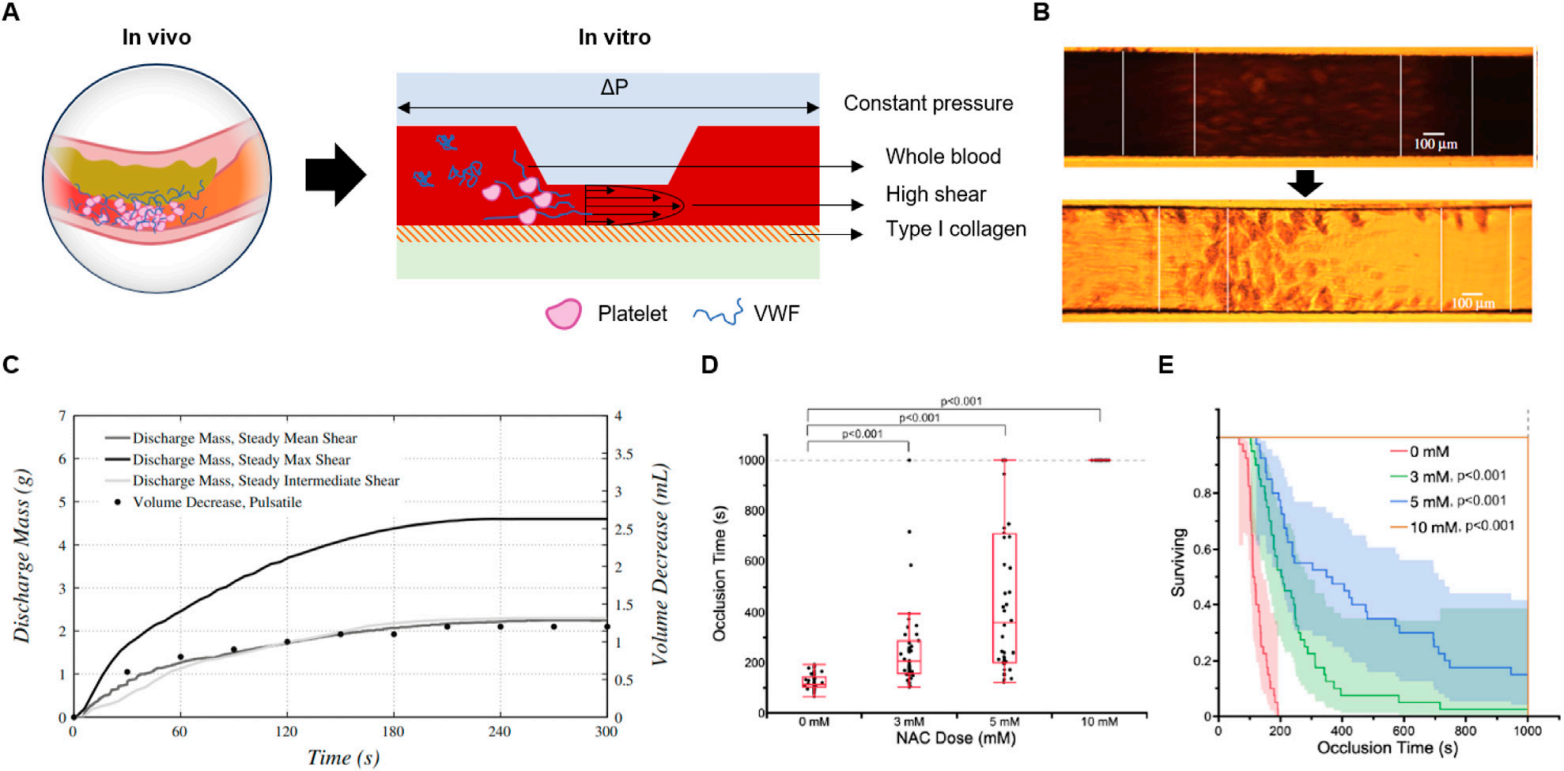
The principle and design of microfluidic POC PFTs. **(A)** Schematic illustrating the reproduction of high-shear thrombosis in *in vitro* microfluidic platforms, mimicking arterial conditions. **(B)** Optical microscopy images capturing SIPA-induced occlusion within microchannels, demonstrating the thrombus growth process. Reprinted from Rooij et al. (2020), Copyright 2020, with permission from the Royal Society. **(C)** Graph showing discharge mass over time as SIPA progresses, correlating with thrombus formation. Reprinted from Casa and Ku. (2014a), Copyright 2014, with permission from Springer Nature. **(D, E)** Application of hemodynamic microfluidic POC PFTs in the development of antiplatelet therapies: **(D)** Occlusion time at various NAC doses, demonstrating dose-dependent effects, and **(E)** Kaplan-Meier survival plot versus occlusion time, assessing the therapeutic efficacy of antiplatelet agents. Reprinted from Bresette et al. (2024), Copyright 2023, with permission from Wolters Kluwer Health.

Recent advances in microfluidic technology have harnessed key determinants of arterial thrombosis by designing microchannels that replicate precise shear conditions and thrombotic surfaces, offering a quick and accurate assessment of platelet function under physiologically relevant, high-shear environments. Figure 3A presents a schematic of a representative microfluidic platform for evaluating platelet function. In these systems, whole blood is perfused through a microchannel with a stenotic region under constant pressure, closely mimicking *in vivo* conditions. Wall shear rate (WSR) is a crucial parameter that influences high shear thrombus growth rate. (Mehrabadi et al., 2016). In parallel plate flow, WSR is derived as the velocity gradient perpendicular to the channel wall and is mathematically expressed as:

$$WSR=6Q/wh^{2}$$

where Q is the volumetric flow rate, w is the width of the channel, and h is the channel height. This equation demonstrates that the shear rate increases with higher flow rates and smaller channel dimensions, particularly the height of the channel. By precisely adjusting these parameters, microfluidic devices can replicate the range of shear rates found in various vascular environments. In constant pressure flow setups, the flow rate is controlled by applying a specific pressure gradient, while the channel geometry ensures that the desired shear conditions are maintained (Figure 3B) (van Rooij et al., 2020). This flexibility in controlling wall shear rate makes microfluidics an ideal platform for studying shear-dependent processes such as thrombosis and platelet aggregation (van Rooij et al., 2020) (Mehrabadi et al., 2016).

In a typical microfluidic system, two types of flow resistors are present: a constant resistor, usually formed by the tubing connected to the chip, and a variable resistor within the microfluidic channel, where resistance increases as the thrombus grows. Under constant pressure conditions, as the thrombus enlarges and increases channel resistance, the flow rate naturally decreases, producing a smooth discharge mass-time curve that closely mimics physiological blood flow dynamics (Figure 3C) (Casa and Ku, 2014a). Constant pressure flow may better-suited for replicating *in vivo* hemodynamic conditions and obtaining accurate diagnostic results related to thrombus growth.

Targeting pathological shear rates that are uniquely present at sites of thrombus development using “smart” therapeutics can be highly effective in preventing total vessel occlusion, significantly improving the efficacy and safety profile of anti-thrombotic therapy. Bresette et al. assessed the efficacy of N-acetyl cysteine (NAC) as an antithrombotic therapy (Figures 3D, E) (Bresette et al., 2024). They demonstrated a significant reduction in thrombus formation following treatment with NAC, both *in vitro* and *in vivo*. At a higher concentration of 10 mmol L^-1^, NAC completely inhibited occlusive clot formation, and no visible macroscopic platelet aggregation was observed (P < 0.001). *In vivo*, a 400 mg kg^-1^ dose of NAC effectively prevented the formation of occlusive clots in mice without significantly affecting tail bleeding times. Mice treated with multiple injections of NAC exhibited a sustained and cumulative effect on clot stability, even after the drug was cleared from circulation (P < 0.001).

**FIGURE 4:**
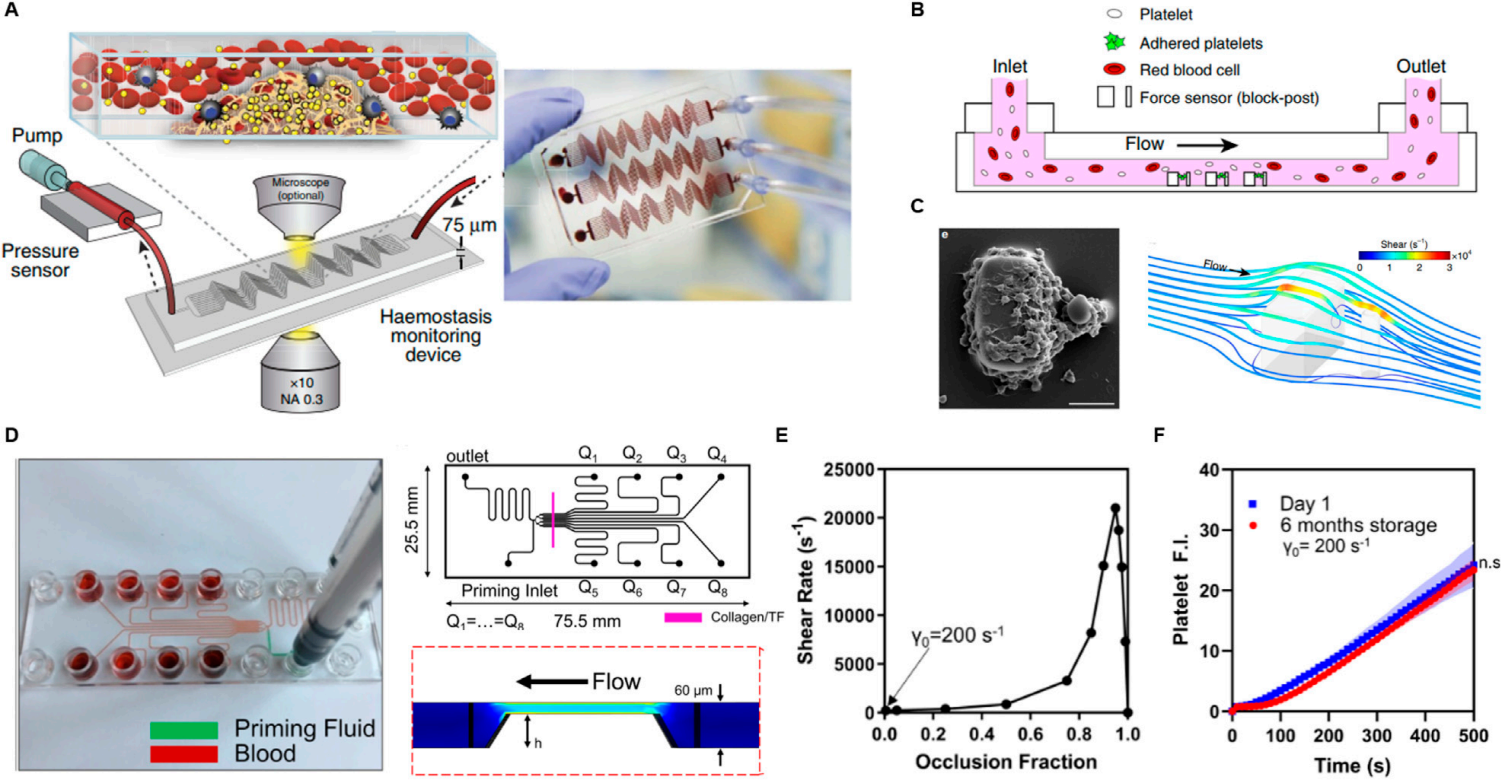
Research progress in microfluidic PFTs. **(A)** Schematic of a hemostasis monitor device and its method of operation, designed to assess platelet function and thrombus formation. Reprinted from Jain et al. (2016), Copyright 2024, with permission from Springer Nature. **(B)** Schematic of a microfluidic device incorporating high shear rates with an array of microblocks to simulate arterial thrombus formation. **(C)** SEM image showing a platelet aggregate formed after 45 s at a shear rate of 8,000 s⁻^1^, alongside a computational fluid dynamics (CFD) simulation visualizing wall shear rates. Reprinted from Ting et al. (2019), Copyright 2024, with permission from Springer Nature. **(D)** Photograph, design diagram, and CFD simulation of an injection-molded, scalable, manufacturable microfluidic device, highlighting its potential for large-scale production. **(E)** Graph showing shear rate as a function of occlusion fraction, illustrating how shear conditions evolve during occlusion. **(F)** Fluorescent intensity of platelets plotted against whole blood perfusion time at day 1 and after 6 months post-manufacturing, assessing the device’s shelf life and performance stability over time. Reprinted from Rossi and Scott (2020), Copyright 2019, with permission from AIP publishing.

Several other innovative microfluidic platforms have been developed, each offering unique advantages for studying thrombosis, platelet function, and antithrombotic therapies. These systems vary in their design, flow control mechanisms, and materials, allowing for a broad range of applications from high-throughput drug screening to more specialized physiological simulations. Jain et al. developed a microfluidic device that simulates stenosed arteriolar vessels, enabling the assessment of blood clotting under pathophysiological flow conditions using small blood sample volumes (Figure 4A) (Jain et al., 2016). This device integrates a mathematical model for clotting analysis, offering *in vitro* measurements of coagulation and platelet function, and has demonstrated real-time monitoring capabilities in pig models of endotoxemia and heparin therapy. The device shows promise for personalized diagnostics and monitoring of antithrombotic therapy, though challenges such as the need for specialized equipment, high cost, and lack of human clinical validation currently limit its broader clinical adoption. Nonetheless, its ability to provide real-time, reliable data in scenarios where standard clotting assays fall short highlights its potential in personalized medicine, particularly for patients using extracorporeal devices or those undergoing anticoagulation therapy.

Ting et al. introduced a microfluidic platform designed to rapidly measure the contractile force of platelets (Figures 4B, C) (Ting et al., 2019). They induced high shear by forming an array of cubic blocks in a channel and observed platelet aggregation on a surface coated with collagen and VWF to simulate thrombotic conditions. Through computational fluid dynamics simulations, they determined that the shear gradients at the blocks ranged from −2.15 × 10⁶ s^−1^·mm^−1^ to 5.74 × 10⁶ s^−1^·mm^−1^. Platelet forces were measured by tracking the deflection of posts over time. Unlike traditional viscoelastic methods that rely on thrombin and fibrin generation, this system directly quantifies platelet forces, providing a more detailed assessment of platelet functionality. The design of a microfluidic channel with a block array is interesting; however, the device does not exclusively assess platelet function related to SIPA, as blood clotting in this system involves both SIPA and coagulation due to localized high shear near the blocks. The complexity of the measurement methods and geometry, along with the reliance on specialized equipment, limits its ability to provide rapid data and ease of interpretation.

Rossi et al. introduced a scalable approach for manufacturing a disposable microfluidic device aimed at rapid platelet function testing, encompassing SIPA, coagulation, and drug response evaluation under whole blood flow (Figure 4D) (Rossi and Scott, 2020). Their work highlights the use of injection molding-based manufacturing for microfluidic systems, offering a scalable, cost-effective alternative to traditional fabrication methods. While polydimethylsiloxane (PDMS) is commonly used for prototyping microfluidic devices due to its ability to replicate intricate flow channel geometries at the micrometer scale and its low-cost mold design capabilities (e.g., via metal machining, 3D printing, or soft lithography), PDMS-based devices present significant limitations in scaling up production using traditional techniques, limiting their transition from bench to market.

In their scalable manufacturing approach, Rossi et al. utilized cyclic olefin copolymer (CoC) plastic components to create microfluidic channels and stenosis regions. The channels were coated with Type I equine fibrillar collagen and tissue factor, forming a 250 μm-wide strip on an optically clear adhesive substrate. As thrombi grew within the channel, the shear rate increased from an initial 200 s⁻^1^ to over 20,000 s⁻^1^, simulating dynamic thrombus formation under physiological conditions (Figure 4E). Thrombus growth and platelet function were monitored by measuring the fluorescence intensity of both platelets and fibrin. Notably, prefabricated microfluidic chips maintained activity when stored at 4°C for up to 6 months, showing no significant difference in platelet or fibrin signals compared to freshly prepared chips (Figure 4F). However, chips stored at room temperature exhibited a significant reduction in platelet signal within 1 week, likely due to degradation of the GPVI binding domains on the collagen. This indicates another device design consideration and challenge regarding the stability of agonists. The progress in manufacturing in this study marks a significant step toward the commercialization of microfluidic POC PFT devices, bridging the gap between bench-scale research and market readiness.

## 5 Challenges and perspectives

We discussed that microfluidic POC PFTs have significant potential to address the limitations of currently used systems by replicating high-shear thrombus growth through the creation of high-shear flow and thrombotic surfaces. However, their development remains in the prototype stage and is often not aligned with the design criteria required to meet clinical demands. These criteria include pathologic shear conditions, rapid test results, ease of use and interpretation, clear and quantified differentiation between normal and abnormal results, and actionable information to guide therapy or transfusion decisions. This section groups these key challenges into i) test section considerations, ii) scalable manufacturing constrains, and iii) human factor design.

### 5.1 Test section considerations

The precise control of flow dynamics in platelet aggregation assays is crucial. Thrombus growth progressively narrows the channel and increases the wall shear rate. Arterial thrombus growth rates are known to peak and decline at approximately 20,000 s⁻^1^ (Mehrabadi et al., 2016). Thus, too low or too high initial wall shear rate decelerates the thrombus growth, leading to longer testing time and larger blood sample amounts, which may not meet the clinical requirements of POC testing. Beyond dictating shear rate, the geometric design of fluidic channels is crucial for forming clots that mimic physiologic SIPA. Smaller channels may emphasize platelet adhesion to surfaces, while larger channels are better suited for assessing platelet-platelet interactions (Casa and Ku, 2014a). A microfluidic channel should not be so small as to become uninterpretable, however, larger channels may significantly increase the amount of blood needed to occlude the channel. The test section where occlusion occurs should have specific geometric features with sufficient length to support stable thrombus formation. The variable diameter and thickness of the membrane in PFA-100 can hinder stable thrombus formation. Likewise, certain PFTs with short needle-shaped stenoses under 50 microns in width fail to support bulk thrombi typically formed in anatomical stenoses with lengths exceeding 200 microns (Nesbitt et al., 2009; Liu et al., 2022).

Next, selecting an appropriate flow type is crucial. Constant flow systems driven by a syringe pump are useful for research on platelet surface adhesion. However, constant flow can introduce significant diagnostic variability in the SIPA test, where the growing thrombus narrows the flow channel. In a continuous flow mode, the growing thrombus rapidly increases the fluid pressure and the shear rate may be orders of magnitude above the pathologic shear condition, leading to noisy spontaneous flow rate fluctuations and reduced diagnostic accuracy (Al et al., 2019; Kaikita et al., 2019). Furthermore, in microfluidic chips with multiple channels, constant flow setups may exacerbate variability. If one channel occludes more rapidly, flow is diverted into the other channels. This increases the flow rate and wall shear rate in the other channels, causing large variations in shear conditions between channels and uneven thrombus growth between channels, ultimately increasing the variability of experimental outcomes. On the other hand, constant pressure conditions avoids from such issues and may be advantageous in reducing outcome variability and multiple flow channels. This is because the flow rate naturally decreases as the thrombus enlarges and increases channel resistance, preventing shear rates from spiking. However, constant pressure may be technically more difficult and complex, requiring a feedback loop including a flow pump, pressure sensor, and a chamber. Pulsatile flow is extremely difficult toimplement in microfluidic platforms and is not necessary for the replication of arterial thrombosis. Pulsatile flow and following oscillatory shear have been shown not to affect high shear thrombosis (Breugel and Heethaar, 1988; High and Ku, 2014b). Thus, simple laminar flow is sufficientwhen designing PFTs.

Maintaining consistent flow dynamics through careful channel design and handling is essential for reliable results. Bubbles are a common and significant issue in achieving high reliability in microfluidic platforms, as they can easily form due to air entrapment during fluid loading, sudden changes in flow rate, or improper sealing of the device, leading to disruptions in fluid dynamics and compromised test accuracy (admin, 2019; Huerre et al., 2014). Even a few small bubbles can significantly affect shear rates and pressure, impacting thrombus formation and outcomes. The chip’s design plays a critical role in preventing bubble formation. Smooth transitions reduce turbulence where bubbles might form. Pre-conditioning fluids, like degassing blood samples, help minimize bubble formation, while passive traps capture any that do occur. Surface treatments, such as hydrophilic coatings, prevent bubbles from adhering to channel walls. Unintended particles, such as dust, also severely impact flow dynamics. When lodged in a stenosis region, a particle can dramatically increase shear rates, causing premature occlusion and diagnostic errors. Similarly, if a circular particle becomes trapped in the flow channel, it creates a new high-shear zone, which acts as an additional thrombus formation site. This depletes platelets and vWF, adds flow resistance, and alters shear rates at the stenosis, delaying occlusion and complicating the test results further.

### 5.2 Scalable manufacturing

In microfluidic platforms, precise and reliable scalable manufacturing of flow channels is challenging, and at the same time, crucial to secure low variability in diagnostic results. Micron-level defects in test section geometry may significantly alter the wall shear rate and thrombus growth rate. According to the predictive model of SIPA, a 10% deviation in channel height increases the occlusion time by 46% (Mehrabadi et al., 2016). The stenosis region width typically ranges from tens to hundreds of micrometers. Assuming a 100-µm test section height, a 10% deviation corresponds to only 10 µm. Thus, the manufacturing tolerance should be within a few micrometers to achieve acceptable variance in diagnostic outcomes. Enlarging the channels may be considered to address this challenge; however, it exponentially increases the amount of blood used per test and creates a consequential hurdle for practical use as POC.

Many prototypes developed in research settings feature complex channel geometries that are difficult to reproduce consistently on a mass-production scale. Simplifying these designs, without sacrificing functionality, will be critical to ensuring that microfluidic devices can be manufactured at scale while maintaining high performance. Furthermore, the materials used in prototype devices often lack the robustness required for long-term clinical use and scalability. Materials must be carefully selected to ensure biocompatibility, durability, and cost-effectiveness, all while facilitating large-scale manufacturing processes such as injection molding or roll-to-roll fabrication. Addressing these challenges is pivotal for transforming microfluidic POC PFTs from academic innovations to practical tools in clinical settings.

Proper constituent materials should be explored for scalable manufacturing. PDMS remains one of the most commonly used materials for fabricating microfluidic flow channels due to its flexibility and ease of use in prototyping. However, PDMS is not suitable for large-scale commercial or clinical applications. The challenges stem from its limited scalability in manufacturing, primarily because the casting process is time-consuming, and molds can only be reused a finite number of times before degradation (Rossi and Scott, 2020). Glass, a widely used substrate for microfluidic POCs, also has its limitations (Wlodarczyk et al., 2018). Although it provides excellent optical clarity and chemical resistance, glass is a poor substrate for applications requiring the deposition of biomolecules such as type I collagen or other protein-based agonists (Siddique et al., 2021; Woodcock et al., 2005; Li et al., 2009). Its hydrophilic surface interferes with collagen deposition, reducing assay performance. Additionally, glass is brittle and prone to breakage, making it difficult to integrate with other components like connectors or sensors, which are essential for a fully functional device. These characteristics make glass impractical for high-throughput manufacturing, as its fragility increases the likelihood of production failure.

As alternatives, thermoplastic materials such as CoC and polyethylene have gained significant attention and several enterprises provide on-demand manufacturing services (Agha et al., 2023; Guan et al., 2022). These materials are hydrophobic, allowing for better deposition of collagen and other proteins necessary for diagnostic assays. Furthermore, CoC and polyethylene are thermoplastics, meaning they can be softened and reshaped multiple times, which is critical for scalable, cost-effective manufacturing (Roy et al., 2012; Leech, 2009). Their potential for use in large-scale production processes such as injection molding ensures that devices made from these materials can be produced in high volumes with consistent quality at a lower cost.

To meet the demand for scalable manufacturing, various fabrication techniques should be explored. Injection molding is widely regarded as one of the most promising methods for mass production of microfluidic devices (Rossi and Scott, 2020). It offers high repeatability and precision, which is essential for creating the small channels required in these devices. However, the initial costs for designing and producing molds with micrometer-scale features are substantial, making this approach less attractive for small-scale operations or research labs (Becker, 2009). Nevertheless, as demand increases and the market grows, these initial costs can be amortized, making injection molding a more viable option for large-scale production.

Micro injection molding is another technique under development, which holds the promise of overcoming some of the limitations of traditional injection molding. This process is designed for even smaller, more intricate features, but it requires further research to optimize its reliability and precision for commercial applications. 3D printing, although a rapidly advancing technology, is not yet fully suited for low-cost, high-volume manufacturing of microfluidic devices. While it offers flexibility in design and the ability to rapidly produce prototypes, the technology is currently limited by its precision and speed. Small deviations in channel geometry, often introduced by 3D printing processes, can significantly affect device performance, making it challenging to ensure the reliability and repeatability needed for scalable production. Moreover, the costs associated with producing large batches of chips via 3D printing remain high, making it less practical for large-scale manufacturing.

Another challenge lies in the application and stability of agonists, which are critical for initiating platelet responses during testing. Agonists such as type I collagen, ADP, and tissue factor are proteins prone to degradation and denaturation even under ambient conditions (Leikina et al., 2002; Li et al., 2009; Griffin et al., 2019). For example, type I collagen starts to unfold and denature around 40°C, and both humidity and dryness can negatively impact its stability. Additionally, its fibrillogenesis can result in uneven surface coverage, influenced by factors like substrate hydrophobicity and solution acidity. This inconsistency in surface coverage leads to irregular vWF adsorption and platelet aggregate capture, which affects the test’s accuracy and reproducibility (Liu et al., 2022). To address these challenges, robust coating techniques are needed to ensure uniform surface coverage. Investigating optimal storage methods and determining shelf-life is also crucial. Advanced surface treatments or chemical stabilizers could be explored to maintain consistent agonist activity and ensure reliable performance across multiple testing cycles. To maintain the activity of agonists, many commercially viable PFTs provide instructions to store them in a refrigerator at a low temperature that ranges from 0°C to 8°C.

### 5.3 Human factor for design

The clinical environment where POC devices are used can be highly variable compared to a research lab or laboratory testings, leading to diagnostic errors. Therefore, the robustness and simplicity is desired in a POC (Farmer et al., 2022). The FDA defines human factors as the integration of “the knowledge about human behavior, abilities, limitations, and other characteristics of medical device users to the design of medical devices … to enhance and demonstrate safe and effective use” (Food and Drug Administration, 2016). Technology developers can apply human factors principles to optimize the implementation of POC technologies, allowing for increased access to diagnostic testing and ultimately for a positive impact on patient care and public health.

For POC PFTs to provide actionable clinical information, they must integrate not only optimized flow dynamics but also clear, easily interpretable endpoints that reflect direct physiological responses to agonists, such as antiplatelet therapies. For example, T-TAS monitors thrombus formation via upstream pressure changes while under flow control, with the area under the curve (AUC) used as a measure of thrombogenicity. However, AUC still needs to be interpreted into a score that reflects clinical platelet function, whether normal or abnormal. Metrics such as occlusion end volume may provide clinicians with a more intuitive and straightforward outcome for assessing platelet function, enabling quicker and more informed decisions.

Next, POC PFTs should provide reliable metrics in a short time. Rapid results are crucial in emergency settings, such as trauma, surgery, or heart attack, where a patient’s SIPA status directly influences treatment choices. These devices must balance speed with precision to ensure an accurate assessment of platelet reactivity without sacrificing reliability, which is especially important in contexts with high stakes for patient outcomes. This need is also linked to another requirement for easy preparation and loading of whole blood samples.

Next, integrating automated redundant systems is essential to guarantee continuous functionality and facilitate real-time issue identification for mitigation of human intervention. These redundant components ensure that, even in the case of minor malfunctions, the device can continue operating effectively without downtime. Furthermore, key system elements should be designed for ease of access and replacement, allowing quick checks and component swaps as needed. This approach maintains the POC’s assessment capabilities, supporting consistent reliability and minimizing disruptions to clinical workflows.

## 6 Conclusion

Microfluidic POC PFTs have significant promise in improving platelet function testing, especially through the replication of high-shear thrombotic conditions. However, substantial challenges remain in ensuring their reliability and clinical relevance. The complexity of flow dynamics, particularly the replication of appropriate shear rates, is critical for accurately assessing platelet function and minimizing test times, yet many current devices fail to meet these needs. In addition to flow dynamics, consistent and scalable manufacturing techniques are essential to transition these devices from research settings to widespread clinical use. Challenges in replicating complex microchannel geometries and ensuring uniform application of agonists, as well as practical issues such as bubble formation and unintended large particles in collected whole blood, must be addressed to maintain accuracy and reliability. Advances in fabrication techniques, such as the use of thermoplastics like CoC and polyethylene, offer potential solutions, enabling better material properties and high-volume production. Addressing these limitations through a combination of optimized flow dynamics, reliable materials, and scalable manufacturing processes will be crucial in advancing microfluidic POC PFTs from academic prototypes to practical clinical tools. By improving both the diagnostic accuracy and scalability of these devices, they have the potential to significantly enhance the timeliness and precision of platelet function assessments, ultimately leading to better patient outcomes in clinical settings.
